# A novel method of synchronous closure for endoscopic full-thickness resection of exophytic gastrointestinal stromal tumors

**DOI:** 10.1055/a-2665-7935

**Published:** 2025-08-22

**Authors:** Jingjing Jiang, Qianyun Ma, Liansong Ye, Rui Sun, Zhongshang Sun, Lei Xu, Feng Pan

**Affiliations:** 1 91596 Department of Gastroenterology, The Affiliated Huai′an No. 1 People′s Hospital of Nanjing Medical University, Huai'an, China; 2 12579 School of Medicine, Southeast University, Nanjing, China; 3 34753 Department of Gastroenterology and Hepatology, West China Hospital, Sichuan University, Chengdu, China

Gastrointestinal stromal tumors (GISTs) are the most common mesenchymal gastrointestinal tumors with malignant potential 1 2 . Endoscopic full-thickness resection (EFTR) is the primary treatment for exophytic GISTs 3 4 . However, there are problems such as large perforation wounds, long exposure time, leading to abdominal cavity contamination and difficulty in suturing. We first proposed a novel synchronous closure technique during EFTR for exophytic GISTs ( Video 1 ).

An innovative method of synchronous closure was applied to resect the exophytic gastrointestinal stromal tumors under endoscopy.Video 1

A 68-year-old female was found to have a 2.0-cm submucosal tumor approximately in gastric fundus during a routine gastroscopy ( Fig. 1 **a** ). Endoscopic ultrasound revealed a muscularis propria-origin hypoechoic lesion, suggesting an exophytic GIST ( Fig. 1 **b** ). Initially, after circumferential incision of the mucosa, we fixed the oral side of the lesion with a metal clip to prevent tumor from sliding during the procedure ( Fig. 2 **a** ). Another clip with a traction line was applied to pull the tumor toward the gastro lumen side continuously ( Fig. 2 **b** ). The anal side was fully incised until full-thickness perforation ( Fig. 2 **c** ). A clip was immediately applied for preclosure and as a preemptive hemostatic measure minimizing perforation time and leakage risk ( Fig. 2 **d** ). The dissection of the tumor was performed, and clips were synchronously and progressively used to close the wound, until the tumor was stripped ( Fig. 2 **e** ). Finally, the wound was completely closed easily ( Fig. 2 **f** ). The entire procedure took 12 minutes. The process diagram is shown in Fig. 3 . The postoperative pathology confirmed a low-risk GIST. This method prevented accidental wound enlargement that would necessitate other complex suture methods, such as purse-string suture and intraoperative puncture and deflation of the abdominal cavity. The operation time was significantly shortened and could reduce the risk of tumor implantation.

**Fig. 1:**
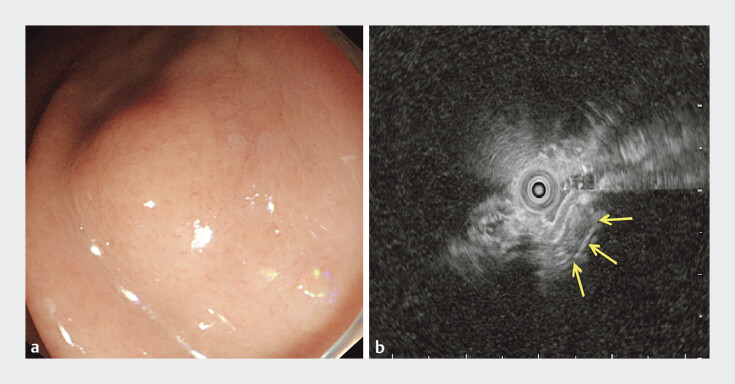
The endoscopic appearance of the gastrointestinal stromal tumor (GIST). **a** The tumor manifests as a submucosal mass with smooth margins and normal overlying mucosa under white light endoscopy. **b** The tumor shows the low-echoic feature of muscularis propria-origin on endoscopic ultrasound, suggesting an exophytic GIST.

**Fig. 2:**
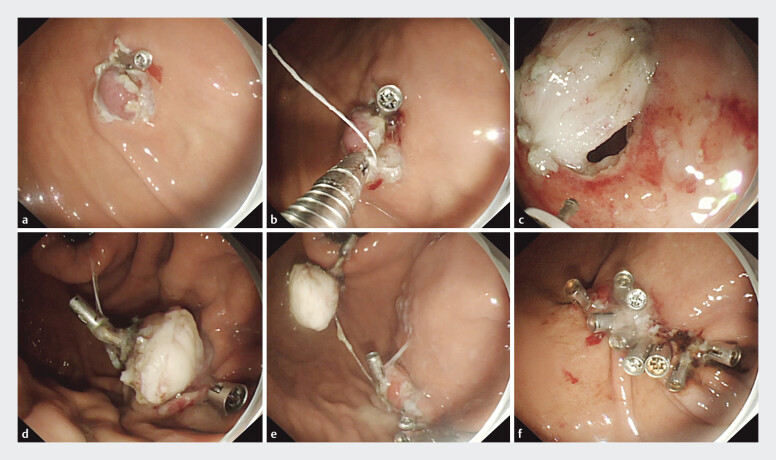
Images of the endoscopic surgery process with synchronous closure. a After incising the oral side mucosa, **a** metal clip was applied to prevent the tumor from sliding during the procedure. **b** Another metal clip with a traction line was fixed onto the surface of the tumor to pull the tumor toward the gastro lumen side continuously. **c** By dragging the traction line, the tumor was pulled toward the gastric cavity, and the anal side mucosa was then incised. **d** A metal clip was immediately applied to preclose the newly occurred perforation, which also restricted the movement of the tumor. **e** While dissecting the tumor, metal clips were synchronously used to close the wound until the tumor was completely resected. **f** The surgical incision, which had been basically closed by metal clips, was then subjected to final management.

**Fig. 3:**
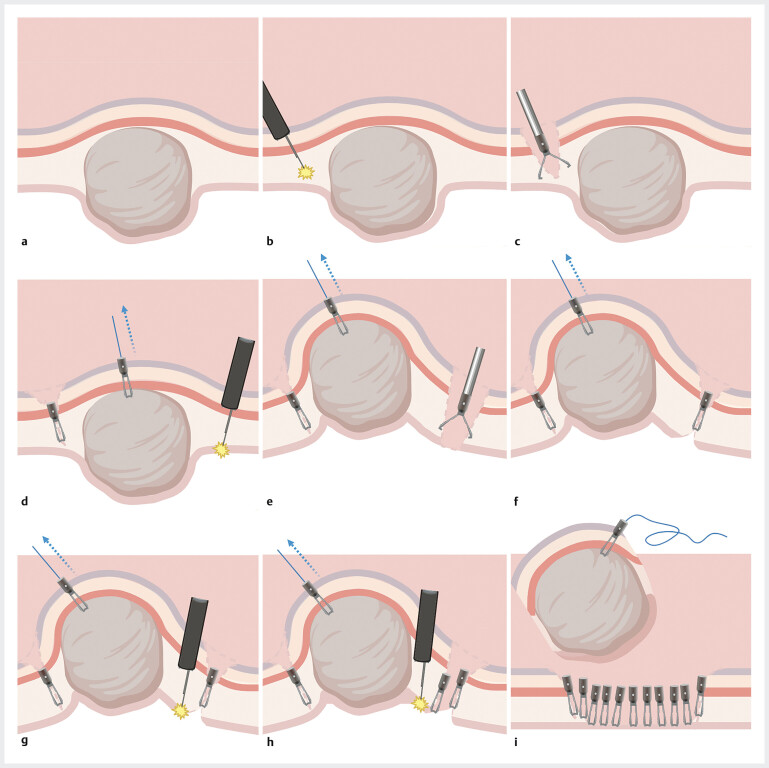
A schematic diagram of the process of resecting gastrointestinal stromal tumors using the synchronous closure method under endoscopy. **a** The submucosal tumor, originating from the muscularis propria, protrudes into the gastric cavity. **b** The oral side mucosa of the tumor was incised. **c** Metal clips were used to fix the incised mucosa, thereby restricting the displacement of the tumor. **d** A metal clip clamp and pull the tumor toward the gastro lumen side with a traction line. **e** The anal side mucosa was then incised and a perforation occurred in the anal side mucosa. **f** The perforation was immediately preclosed with a single metal clip. **g** While traction was applied, the tumor was further dissected. **h** The dissection of the tumor was performed with synchronous closure. **i** The tumor was completely resected, and the wound was closed.

According to our experience, the novel technique of synchronous closure during EFR is simple and highly effective for endoscopic resection of exophytic GIST. Further studies with large sample sizes are needed to confirm its value.

Endoscopy_UCTN_Code_TTT_1AO_2AI

## Notes

Endoscopy E-Videos https://eref.thieme.de/e-videos
*E-Videos* is an open access online section of the journal *Endoscopy* , reporting on interesting cases and new techniques in gastroenterological endoscopy. All papers include a high-quality video and are published with a Creative Commons CC-BY license. Endoscopy E-Videos qualify for HINARI discounts and waivers and eligibility is automatically checked during the submission process. We grant 100% waivers to articles whose corresponding authors are based in Group A countries and 50% waivers to those who are based in Group B countries as classified by Research4Life (see: https://www.research4life.org/access/eligibility/ ). This section has its own submission website at https://mc.manuscriptcentral.com/e-videos .
